# Reductions in inpatient and outpatient mental health care in germany during the first year of the COVID-19 pandemic – What can we learn for a better crisis preparedness?

**DOI:** 10.1007/s00406-024-01909-6

**Published:** 2024-10-02

**Authors:** Fabian Baum, Jochen Schmitt, Oliver Nagel, Josephine Jacob, Martin Seifert, Kristina Adorjan, Oliver Tüscher, Klaus Lieb, Lars Peer Hölzel, Hauke Felix Wiegand

**Affiliations:** 1 InGef - Institute for Applied Health Research Berlin GmbH, Berlin, Germany; 2 Department of Psychiatry and Psychotherapy, LMU University Hospital, Munich, Germany; 3 Department of Psychiatry and Psychotherapy, University of Bern, Bern, Switzerland; 4 Department of Psychiatry and Psychotherapy, University Medical Center of the Johannes Gutenberg- University Mainz, Mainz, Germany; 5 Center for Evidence-Based Health Care (ZEGV), Faculty of Medicine Carl Gustav Carus, University Hospital Dresden, Technische Universität Dresden, Dresden, Germany; 6 Department of Psychiatry, Psychotherapy and Psychosomatic Medicine, University Medicine Halle, Martin-Luther University Halle-Wittenberg, Halle, Germany; 7 Oberberg Parkklinik Wiesbaden Schlangenbad, Schlangenbad, Germany

**Keywords:** Claims data, Mental health care, Statutory health insurance, Inpatient treatment, Outpatient treatment

## Abstract

Background: During the COVID-19 pandemic, reports from several European mental health care systems hinted at important changes in utilization. So far, no study examined changes in utilization in the German mental health care inpatient and outpatient mental health care system comprehensively. Methods: This longitudinal observational study used claims data from two major German statutory health insurances, AOK PLUS and BKK, covering 162,905 inpatients and 2,131,186 outpatients with mental disorders nationwide. We analyzed changes in inpatient and outpatient mental health service utilization over the course of the first two lockdown phases (LDPs) of the pandemic in 2020 compared to a pre-COVID-19 reference period dating from March 2019 to February 2020 using a time series forecast model. Results: We observed significant decreases in the number of inpatient hospital admissions by 24–28% compared to the reference period. Day clinic admissions were even further reduced by 44–61%. Length of stay was significantly decreased for day clinic care but not for inpatient care. In the outpatient sector, the data showed a significant reduction in the number of incident outpatient diagnoses. Conclusion: Indirect evidence regarding the consequences of the reductions in both the inpatient and outpatient sector of care described in this study is ambiguous and direct evidence on treatment outcomes and quality of trans-sectoral mental healthcare is sparse. In line with WHO and OECD we propose a comprehensive mental health system surveillance to prepare for a better oversight and thereby a better resilience during future global major disruptions.

**Supplementary Information:**

The online version contains supplementary material available at 10.1007/s00406-024-01909-6.

## Introduction

The COVID-19 pandemic was a disruptive event for health care systems worldwide that had severe effects on mental health service utilization as well. Capacity-limiting infection protection measures were implemented, capacities for patients with severe mental illness and co-occurring SARS-CoV-2 infection were created, and service user’s utilization behavior might have been influenced by fears of infection in health service institutions and socioeconomic challenges like social isolation and economic insecurity [1–5]. Furthermore, political measures like incentives for keeping hospital capacities unoccupied might have impacted healthcare provision [6].

The WHO and the OECD highlighted the necessity to improve resilience and crisis preparedness of health care systems to be able to maintain essential health care for vulnerable populations during major disruptions [7, 8], what should include those with mental illness [9]. A first necessary step for a better crisis preparedness is to understand the extent of change and to identify the most affected sectors and patient groups during the COVID-19 pandemic.

A systematic review on the pandemic’s initial lockdown phases showed overall decreases in inpatient mental health service admissions as well as reduced outpatient service utilization [10]. However, for many regions of Europe so far no comprehensive studies exist: For Germany, surveys of inpatient departments and routine data studies of two regional inpatient provider networks reported reductions of inpatient treatment utilization to 60–80% and day clinic treatment to 50-70% of the 2019 levels. These reductions seemed to have gone along with relative increases in urgent and involuntary admissions and coercive measures [1, 11, 12]. For the outpatient system no peer-reviewed studies have been published so far.

Therefore, the goal of this study was to comprehensively and systematically analyze in a large routine data set, which sectors (inpatient services and outpatient psychiatric, psycho-pharmacotherapeutic and psychotherapeutic services) and which patient populations stratified according to pre-defined diagnostic groups of mental disorders were affected by reductions in mental health service utilization in Germany during the first year of the COVID-19 pandemic.

## Methods

### Study design

The study is a longitudinal observational study and was developed as part of the German Network University Medicines (NUM) projects egePan Unimed and PREPARED, focusing on the development, evaluation, and implementation of evidence-based pandemic management and pandemic preparedness. Over a total period from January 2016 to April 2021 (only inpatient data, the time interval for outpatient data was cut off December 2020), all insured persons ≥ 18 years of age (at the time point of their first mental health related service utilization in in the observation period) with mental and behavioral disorders were included in the study. Particular focus was placed on changes in utilization at the time around the first and second lock-down (March to May 2020 and December to February 2021) and the intermediate time between the two lockdowns (July to September 2020) compared to the intervention-free reference period (March 2019 to February 2020).

### Data and outcomes

We used nationwide claims data from two major German statutory health insurances (SHI), AOK PLUS and and BKK (via InGef research database, including mainly company or guild health insurances [13]). The data covers a total of 8.8 million insured individuals. While the AOK Plus data set is limited to patients living in Saxony, the BKK data set comprises patients from all 16 federal states and the sample sizes per federal state roughly matches with their population. In addition to sociodemographic characteristics (age and sex) and vital status (via the date of death), the data include comprehensive information on healthcare utilization in outpatient and inpatient sectors. The data includes diagnoses (according to the International Statistical Classification of Diseases and Related Health Problems - German Modification, ICD-10-GM), procedures (according to the “Operationen-und Prozedurenschluessel,” OPS; German modification of the International Classification of Procedures in Medicine, ICPM), information on outpatient helthcare services (according to “Einheitlicher Bewertungsmassstab,” EBM), and prescribed medications (identified by the International Anatomical Therapeutic Chemical (ATC) Classification and the WHO defined daily doses (DDD) classification).

We defined a set of outcomes based on inpatient, day clinic and outpatient diagnoses according to ICD-10-GM and the guidelines good practice secondary data analysis [GPS, 14] of the German Society for Epidemiology [DGEpi, 15]. For inpatient and day clinic care, these outcomes were: Total Number of inpatient admission, average length of inpatient stay, number of days in standard care and number of days in intensive (psychiatric) care. The last two indicators we used as an indicator for the severity and acuity of the patients in inpatient treatment. They are defined by standardized criteria of severity and acuity and applied because reimbursement for intensive care is higher. Note that data for regular and intensive inpatient treatment was only available in the BKK data set as it is indicated by a specific OPS marker and not to be found in the AOK data set. For outpatient care, outcomes of interest were: Total number of incident diagnoses, number of patients with at least one therapeutic session, as well as the total number of DDDs of psycho-pharmacotherapeutics prescribed, additionally separated into eleven different substance groups identified by their ATC code (please see supplement 1). Whenever possible, we stratified these outcomes for eight predefined diagnostic groups (see Table 1). This was the case for all outcomes of inpatient care as well as for the total number of incident outpatient diagnoses.

**Table 1:** A priori defined diagnostic groups of mental diseases (ICD-10)

F-Code	Diagnostic group
F0	Organic, including symptomatic, mental disorders
F10-19	Mental and behavioral disorders due to psychoactive substance use
F22-29	Schizophrenia, schizotypal and delusional disorder
F30-34	Affective disorders
F40-45	Neurotic, stress-related and somatoform disorders (including anxiety disorders and OCD)
F50	Behavioral syndromes associated with physiological disturbances and physical factors
F60-61	Personality Disorders
all other F-Codes	other

### Statistical analysis and evidence synthesis

We modeled the time series based on an autoregressive forecast approach [16, 17]. This method uses a likelihood-based estimation method for analysis and modeling of count time series following generalized linear models. Negative binomial regression models were fitted on the monthly counts for the period January 1, 2016, to April 30, 2021. All inpatient data was formatted as monthly time-series data, outpatient data was fitted as quarterly time-series data. Patients were the unit of observation, and month (inpatient) or quarter (outpatient) was the unit of analysis. The monthly/quarterly counts were used as the outcome in the models. The time bins of interest were the first two LDPs of the pandemic, namely March to May 2020 and December 2020 until February 2021. Note, that due to data restrictions, in the outpatient data set we could only consider the first quarter of 2021 as the latest time point. In the analysis, we utilized the two nationwide lockdowns as an explicit cut to contrast changes that happened within the LDP compared to the time prior. Using the starting date of the lockdowns gave us the advantage of providing a fixed standardized starting point in time across Germany. Thus, we referenced these two averaged time bins against the reference time window, namely the average over the time span of March 2019 until February 2020. Furthermore, in order to test for recovery effects in the lockdown-free period in the summer of 2020, we also contrasted an according time bin (July to September 2020 for inpatient data and 3rd quarter of 2020 for outpatient data) against the time span of March 2019 until February 2020. For the analysis of inpatient and day clinic care, we additionally applied post-hoc tests for some of the diagnostic clusters, namely F10-19, F20-29, F30-34, and F40-45. The selection of the clusters was based on case numbers. We conducted the analysis using the *tscount* package [18] and the statistical software R v4.0.3 [19]. The evaluation of the SHI routine data sets was carried out in accordance with data protection regulations by the respective authorized analysis units.

## Results

The pooled study sample from the two data sets of statutory health insurance funds (AOK PLUS and BKK) included 162,905 patients in the inpatient sector (N_AOK PLUS_=62,238; N_BKK_=100,667) and 2,131,186 patients in the outpatient sector (N_AOK PLUS_=1,187,782; N_BKK_=943,404, see Table 2). The mean age of the sample in the inpatient sector was 56.3 years, 53.2% (*n* = 86,703) were women. The sample of the outpatient sector had an average age of 49.7 years, 61.1% (*n* = 1,301,545) were female.

**Table 2:** Sample characteristics of the pooled data set used in the analysis

	inpatient	outpatient
*N*	162,905	2,131,186
Mean age (SD)	56.3 (20.1)	49.7 (22.1)
Female (%)	86,703 (53.2)	1,301,545 (61.1)
No. of Patients with outpatient subscriptions (%)	-	876,097 (41.1)
Berlin	5,848 (3.6)	74,668 (3.5)
Brandenburg	3,540 (2.2)	29,102 (1.4)
Saxony Anhalt	1,648 (1)	13,916 (0.7)
Saxony	64,779 (39.8)	1,203,974 (56.5)
Thuringia (Thüringen)	1,771 (1.1)	14,921 (0.7)
Mecklenburg-West Pomerania	1,319 (0.8)	13,766 (0.6)
Schleswig-Holstein	2,817 (1.7)	24,977 (1.2)
Lower Saxony	11,452 (7)	122,435 (5.7)
Hamburg	1,664 (1)	18,739 (0.9)
Bremen	285 (0.2)	3,248 (0.2)
Hessen	6,684 (4.1)	55,397 (2.6)
Rhineland Palatinate	6,575 (4)	44,110 (2.1)
Baden Wurttemberg	10,727 (6.6)	126,218 (5.9)
Saarland	894 (0.5)	7,902 (0.4)
Bavaria	15,883 (9.7)	148,731 (6.9)
North Rhine Westphalia	26,821 (16.5)	227,227 (10.7)
unknown	198 (0.1)	1,855 (0.1)

### Inpatient care

**Fig. 1:**
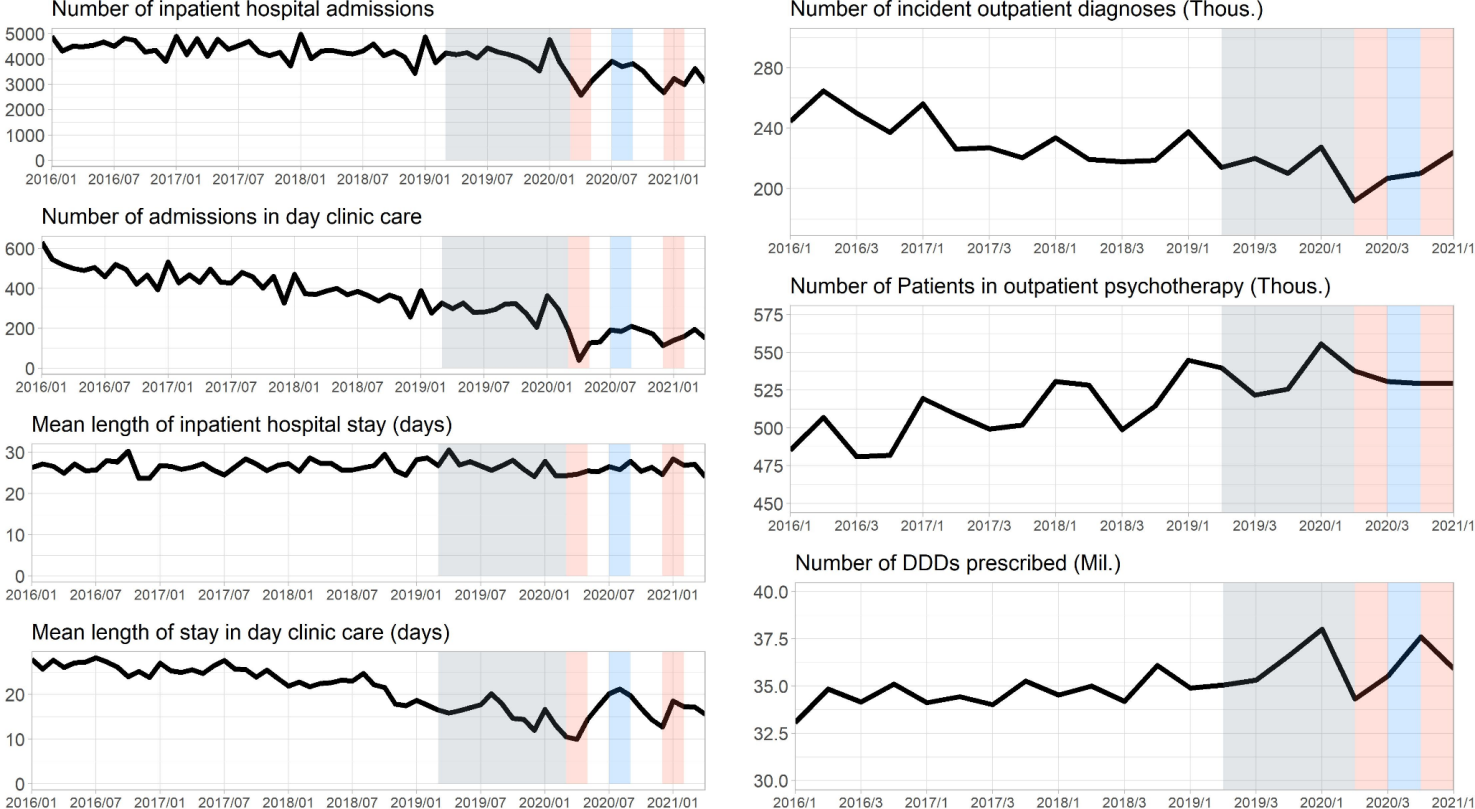
Number of hospital admissions and mean length of hospital stay, number of outpatient incident diagnoses, number of patients with outpatient psychotherapy, and number of DDDs of psychotropic drugs prescribed from March 1st 2019 to April 30st 2021. The months highlighted in red represent the two intervention periods under consideration (1st and 2nd lockdown period), each of which was contrasted with the reference period highlighted in gray. The period highlighted in blue was used to test for recovery effects in the lockdown-free period

**Fig. 2:**
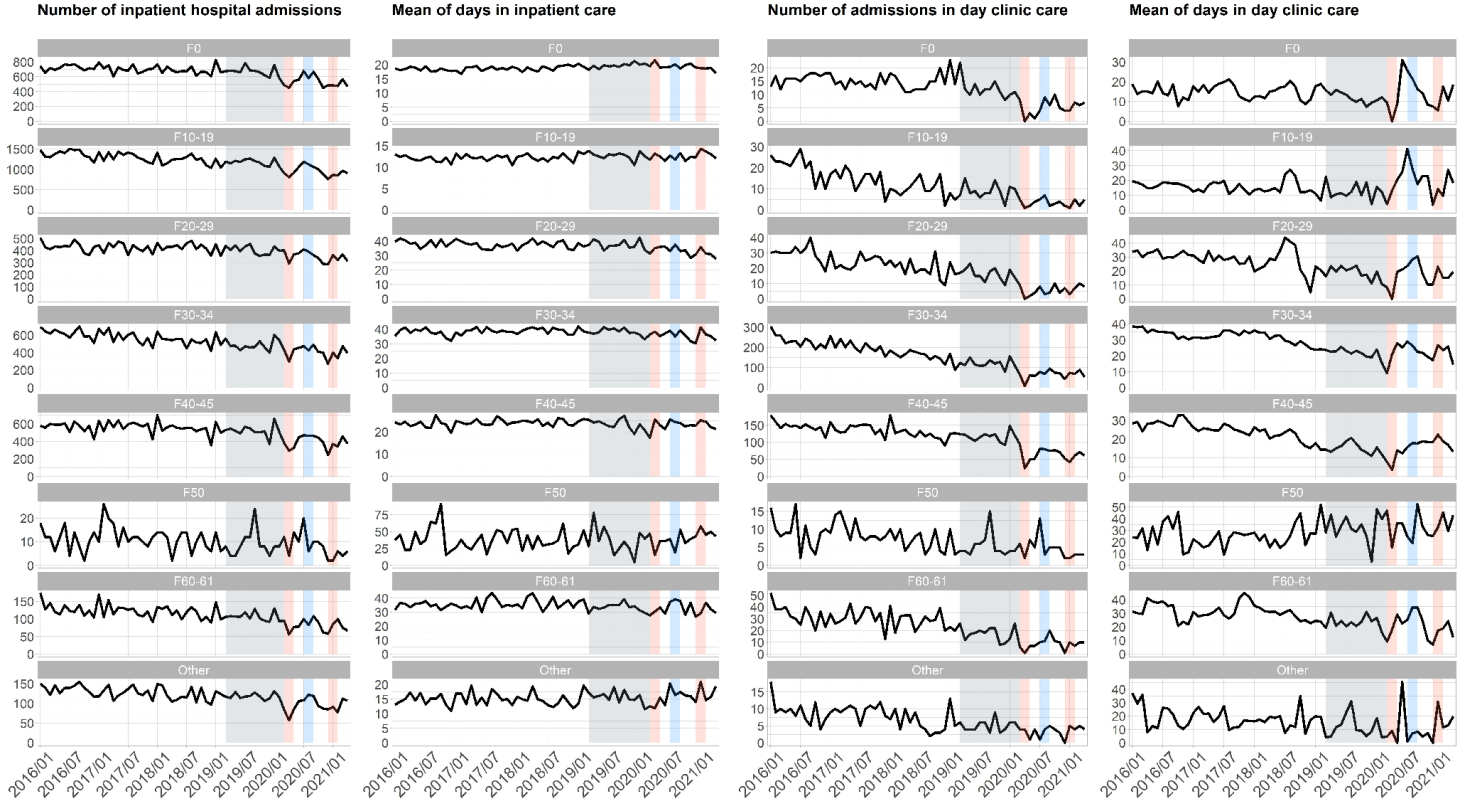
Hospital admissions and mean length of hospital stay from March 1st 2019 to April 30st 2021 by predefined diagnostic clusters. The months highlighted in red represent the two intervention periods under consideration (1st and 2nd lockdown period), each of which was contrasted with the reference period highlighted in gray. The period highlighted in blue was used to test for recovery effects in the lockdown-free period

In comparison to the averaged reference time window (March 2019 to February 2020) we observed significant decreases in the number of inpatient hospital admissions during the first lockdown phase (LDP) by 24% (-1163 admissions), and during the second LDP by 28% (-1175 admissions), respectively (see Fig. 1; Table 3). In both LDPs, this effect was consistent for patients with mental and behavioral disorders due to psychoactive substance use (F10-19) and for patients with anxiety and somatoform disorders (F40-45), in the second LDP for all examined disorder groups. For detailed results stratified by ICD-10 disease groups see Fig. 2; Table 3. Regarding the average length of hospital stay (LOS), the analysis showed no change for both LDPs. However, we observed a significant decrease in the number of days in standard care by 22% during second for LDP while the number of units of intensive care did not show a significant change in any direction. An age-stratification of the number of admissions can be found in Supplement IV. For comparison, Supplement VI plots the psychiatric admissions from our datasets on the same time-axis as general hospital admissions for SARS-CoV-2 infections (data from the German Robert-Koch-Institute, [30]).

**Table 3:** Risk ratios, *p*-values and confidence intervals taken from the time series forecast model

Outcome	Lockdown 03–05/2020				Lockdown-free Period 07–09/2020				Lockdown 12/2020-02/2021			
	estimate	*p*-value	Lower CI	Upper CI	estimate	*p*-value	Lower CI	Upper CI	estimate	*p*-value	Lower CI	Upper CI
**inpatient care**												
Total no. of admissions												
*Overall*	0.76	0.004	0.63	0.91	0.82	0.181	0.62	1.09	0.72	< 0.001	0.66	0.79
*F10-19*	0.75		0.58	0.96	0.96		0.71	1.31	0.62		0.48	0.8
*F20-29*	0.95		0.78	1.16	0.91		0.71	1.17	0.66		0.54	0.82
*F30-34*	0.87		0.73	1.03	0.89		0.61	1.29	0.48		0.35	0.67
*F40-45*	0.71		0.53	0.94	0.78		0.53	1.16	0.41		0.3	0.56
Mean length of stay												
*Overall*	1.06	0.612	0.84	1.34	0.94	0.806	0.617	1.45	0.86	0.48	0.58	1.29
*F10-19*	0.96		0.69	1.34	0.99		0.55	1.78	1		0.55	1.82
*F20-29*	0.83		0.59	1.18	1.02		0.74	1.43	0.81		0.57	1.17
*F30-34*	1.04		0.88	1.23	0.92		0.66	1.31	0.77		0.54	1.11
*F40-45*	0.75		0.47	1.2	1.03		0.69	1.54	0.96		0.63	1.45
Units of standard care*	0.92	0.719	0.6	1.42	0.91	0.697	0.58	1.43	0.78	0.04	0.49	0.92
Units of intensive care*	1.07	0.864	0.48	2.4	1.08	0.862	0.48	2.44	0.86	0.796	0.29	0.252
**day clinic care**												
Total no. of admissions												
*Overall*	0.56	0.022	0.34	0.92	0.75	0.414	0.38	1.49	0.39	< 0.001	0.31	0.49
*F10-19*	0.59		0.23	1.53	0.73		0.17	3.1	0.15		0.03	0.81
*F20-29*	0.49		0.21	1.15	0.2		0.05	0.82	0.31		0.12	0.81
*F30-34*	0.44		0.22	0.89	0.66		0.31	1.42	0.26		0.13	0.53
*F40-45*	0.78		0.56	1.09	0.76		0.43	1.34	0.42		0.26	0.66
Mean length of stay												
*Overall*	0.39	0.015	0.19	0.83	1.06	0.811	0.67	1.7	0.59	0.02	0.32	0.91
*F10-19*	0.29		0.1	0.81	1.8		0.96	3.36	0.23		0.08	0.7
*F20-29*	0.31		0.13	0.72	0.61		0.3	1.26	0.41		0.18	0.92
*F30-34*	0.32		0.17	0.62	1.08		0.71	1.65	0.59		0.36	0.97
*F40-45*	0.38		0.18	0.79	0.82		0.48	1.39	0.86		0.49	1.52
**outpatient care**												
No. of incident cases	0.82	0.001	0.73	0.92	0.91	0.209	0.78	1.06	0.91	0.169	0.81	1.04
No. of patients with psychotherapy	1.01	0.968	0.96	1.04	1.03	0.559	0.94	1.11	0.87	0.493	0.95	1.11
Total sum of DDDs subscribed	0.99	0.693	0.94	1.04	1.02	0.722	0.93	1.1	0.88	0.056	0.96	1.07

### Day clinic care

During the first LDP day-clinic admissions were reduced by 61% (-180 admissions), and during the second LDP by 44% (-162 admissions), respectively, compared to the twelve months prior (see Fig. 1 Table 3). In the first LDP, this effect was mainly driven by reductions in admissions of patients with affective (F30-34) and anxiety and somatoform disorders (F40-45). During second LDP, analogously to the sector of inpatient care, there was a significant drop of hospital admissions for all diagnostic clusters tested separately (see Fig. 2; Table 3).

Regarding the average LOS, the data revealed significant reductions in the aftermath of the first LDP by 3.8 days compared to the average of the reference period. Interestingly, this effect was almost equally distributed across all diagnostic clusters during both LDP with a non-significant effect for F40-45 during the second LDP being the only exception.

### Outpatient care

We observed a significant reduction in psychiatric incident diagnoses by 22% (-26,126 incident diagnoses) in the outpatient sector in the aftermath of the first LDP compared to the reference period (see Fig. 1; Table 3). For the second LDP, the number of incident diagnoses was still reduced by 14% (-7,809 incident diagnoses) but without reaching significance level. The main contributors to the reduction were the diagnostic clusters containing the majority of the patients, such as anxiety and somatoform disorders (F40-45) as well as affective disorders (F30-34, see Supplement II). Changes in clusters with only few patients were not as pronounced. These clusters are presumably characterized by a floor effect as low numbers cannot be reduced any further. An age-stratification of psychiatric incident diagnoses can be found in Supplement V.

Regarding the overall number of patients receiving outpatient psychotherapy we noticed no significant changes for both LDPs (see Table 3). Note that the time line shows a trend of an increasing number of patients in therapy from January 2016 up until the first quarter of 2021 (Fig. 1). Additionally, there was a strong effect of seasonality with a peak in every first quarter of each year.

Prescriptions of psychotropic drugs showed a trend towards an increase over the course of the observation period (see Fig. 1). The first quarter of 2020 showed an initial increase in prescriptions followed by a dip with the onset of the first LDP in the second quarter of 2020, a recovery period afterwards and a similar pattern around the second LDP, both without reaching significance level (see Table 3).

### Analysis of recovery effects in lockdown-free period

**Fig. 3:**
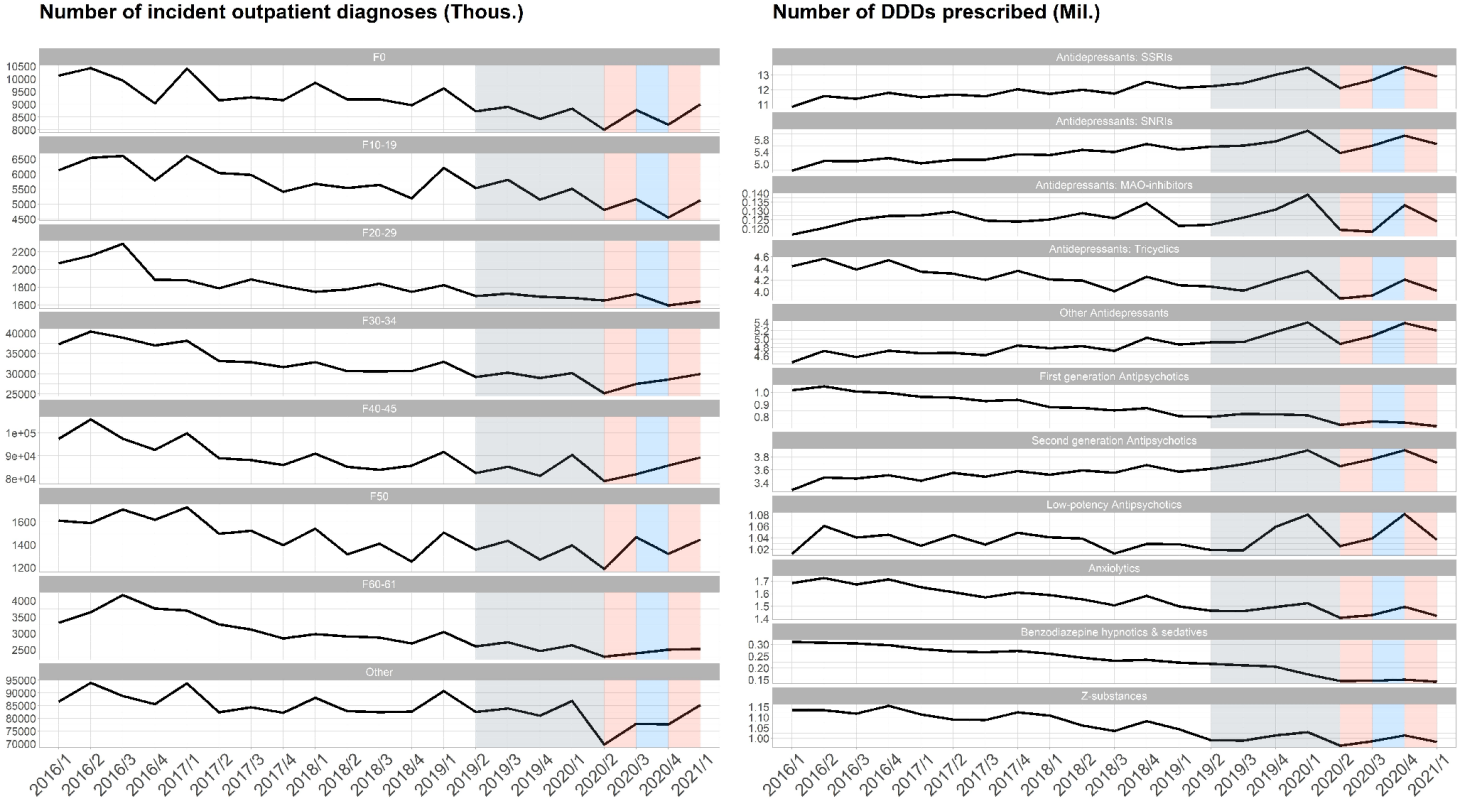
Risk ratios, *p*-values and confidence intervals taken from the time series forecast model. * Please note that the analysis of “units of standard care/units of intensive care” is based on the BKKdata set only

For both the inpatient and outpatient sector, the data showed some reductions in health care provision in the lockdown-free period in summer 2020 in comparison to the averaged reference period (March 2019 to February 2020, see Figs. 1 and 3, and Table 2). However, most of the differences between the time bins tested did not exceed significance threshold anymore.

## Discussion

This study in a large nation-wide claims dataset showed significant reductions in utilization of mental health services in Germany during the first two lockdown phases of the COVID-19 pandemic, foremost in the inpatient and day clinic care sectors and in outpatient incident diagnoses. Prescriptions of psychopharmacological medications and outpatient psychotherapy provision remained stable. In the period between the two lockdown phases, no complete recovery of utilization was observed.

### Inpatient system changes

Mental health inpatient care admissions were significantly reduced, in line with results from other regions of Europe and reports from local German provider networks [1, 5, 10–12, 20]. However, specific diagnostic groups were affected to different degrees: The first lockdown phase showed stistically significant reductions for substance use and anxiety disorders; the second lockdown phase for all diagnostic groups but they were more pronounced for e.g. substance-use, anxiety, obsessive-compulsive and stress-associated disorders or affective disorders but to a lesser degree psychotic disorders. Reduced indicators of standard care but unchanged indicators of psychiatric intensive care are in line with reports of relative increases in more acute cases during the lockdown phases [5, 10, 12]. The day clinic care setting exhibited not only significantly reduced admission numbers in both LDPs but additionally significantly reduced length of stay in the first lockdown phase. Thus, this less life-disrupting and more integrative treatment mode was even more impacted by service reductions.

The reasons for these significant reductions cannot be found out by routine data studies alone. Results of surveys of psychiatric inpatient institutions and office-based outpatient psychiatrists suggested that reduced demand by patients and a lack of staff played minor roles. Mainly, institutions themselves seemed to have changed their admission policies for creating capacities for comorbid, severely mentally ill and infectious patients, and for hygiene and social distancing measures. In the second LDP premature discharges of patients with SARS-CoV-2 infections might have played a role, too. Additionally, financial incentives that were thought to provide capacities for SARS-CoV-2-patients might have been an important factor [5, 21]. Unfortunately, studies of user perspectives are lacking, but the existing results hint at a reduced healthcare provision being a significant factor. This raises questions, if and to what degree these patients were treated by the outpatient system instead or if the reductions resulted in an underprovision of services for people with mental disorders or – as one might argue because of Germanys unique large share of inpatient mental health care – in a normalization of a previous overprovision.

### Outpatient system changes

Outpatient care showed no significant change in the number of patients in psychotherapy and prescriptions of psychotropic drugs. Patients “stocking” prescriptions in order to be prepared for access problems can explain probably slight increases in prescriptions immediately before and small dips during the lockdowns. However, the data showed indications neither for supply gaps nor for an overall increased demand during the first year of the pandemic. In the United Kingdom drops in prescriptions for antidepressants were reported for the first lockdown [22], while psychotherapy services were rarely examined on a national level within Europe. Psychotherapy service provision in one British regional network did not change significantly during 2020 [23].

Significant reductions within the outpatient system were found in the number of incident diagnoses. In line with these results, a survey of outpatient psychiatrists reported maintenance (partly by telemedicine) of offerings for known but reductions for new patients due to capacity restraints by e.g. social distancing measures [21]. Additionally, some outpatient psychiatrists reported a slightly reduced demand in the first lockdown phase, but increased demand in later phases of the pandemic due to catch-up effects, social isolation and economic hardships [21]. However, (financial) caps limit the outpatient system’s ability to provide large capacity increases. Taken together, patients impacted by reductions in inpatient and day clinic services were probably not to a large degree absorbed by outpatient system offerings. However, as no individual patient’s treatment sequences were examined, the currently available evidence cannot definitely answer this question.

### Consequences of reduced mental care services

Due to this lack of studies of individual patient’s treatment sequences and outcomes it cannot be answered neither, if the observed reductions in mental health service utilization let to negative consequences. Indirect evidence is inconclusive: Surveyed psychiatric inpatient departments and outpatient psychiatrists reported exacerbations, contact breakdowns, a lack of integration into the patients’ living environment, and suicide attempts and saw them – without proven causality – linked to reduced inpatient capacities and insufficient outpatient treatment alternatives [5, 21]. Pandemic-related reductions in maintenance electroconvulsive therapies resulted in exacerbations [24]. However, no general increase in suicide mortality was reported for neither Germany nor Europe for the first year of the pandemic [25, 26], but longterm results are lacking, some departments reported increases in suicidality for certain disease groups [27] and no comprehensive statistics on suicide attempts exist.

### Strengths and limitations

This study utilizes a large set of claims data covering a total of 8.8 million insured individuals across Germany, however, with a bias towards the region of Saxony. Nonetheless, since the majority of the results were also confirmed in the subset of the BKK data that is representative for Germany [40] (see Supplement III), the results can be generalized to the whole of Germany. Claims data can offer complete and unbiased information on health care utilization and provision [14, 28]. However, it is restricted to broad indicators only and does not allow to distinguish between changes in utilization (in a narrow sense) and provision. The data itself allows no inference about the causes of changes in health care utilization. Additionally, while claims data offers much information on the provider level, it lacks information on the user perspective. Finally, our study covers only the first year of the pandemic and follow-up studies on later changes and possible catch-up effects would be of great interest.

### Conclusion: A call for a mental health system surveillance

During the first year of the pandemic significant reductions in mental health care service utilization took place, probably mostly as a consequence of (in the light of the imminent threads of COVID-19 well-intentioned) changes in political guidance, necessary hygiene measures, and financial incentives. Some studies suggest important negative consequences of these reductions, but due to a lack of a systematic monitoring or studies of trans-sectoral treatment sequences and routine treatment outcomes, no final conclusion can be drawn.

When during the crisis of the pandemic rapid decisions like e.g. changes in financial incentives and hygienic isolation measures had to be made without an evidence base it was impossible to guide these decisions by relevant up-to-date data and to monitor their effects transparently and in a timely manner – neither in Germany nor in many other regions of Europe. Infrastructures for monitoring and oversight are among the central recommendations for resilient health care systems by the WHO and OECD [7, 8, 29]. We therefore propose that a transparent *public mental health system surveillance* is needed, including indicators of trans-sectoral treatment-sequences and routine treatment outcomes. Indicators like those in this study could be the nucleus for such a comprehensive surveillance that could contribute to a better crisis preparedness and a more resilient mental health care systems in Europe [8].

## Electronic supplementary material

Below is the link to the electronic supplementary material.

Supplementary Material 1

Supplementary Material 2

Supplementary Material 3

Supplementary Material 4

Supplementary Material 5

Supplementary Material 6

Supplementary Material 7

## Data Availability

The data used in this study is owned provided by two German SHI funds. This data is not publicly available as it contains highly sensitive data on health care utilization for the individuals insured at these SHIs. The data was able to be published only under special license for the current study.
