# Supplementary material for: Reductions in inpatient and outpatient mental health care in germany during the first year of the COVID-19 pandemic – What can we learn for a better crisis preparedness?
Source: Eur Arch Psychiatry Clin Neurosci. 2024 Oct 2;274(8):2037–46. doi: 10.1007/s00406-024-01909-6 (PMC11579190; doi:10.1007/s00406-024-01909-6)
Supplement: Supplementary file 1 — Supplementary Material 1 [file 406_2024_1909_MOESM1_ESM.docx]

1. **Supplement Table**

A priori defined substance groups

| **Substance Group** | **ATC code** | **Substance** |
| --- | --- | --- |
| **Antidepressants: SSRIs** | N06AB03 | Fluoxetine |
|  | N06AB04 | Citalopram |
|  | N06AB05 | Paroxetine |
|  | N06AB06 | Sertraline |
|  | N06AB08 | Fluvoxamine |
|  | N06AB10 | Escitalopram |
| **Antidepressants: SNRIs** | N06AX16 | Venlafaxine |
|  | N06AX17 | Milnacipran |
|  | N06AX21 | Duloxetine |
| **Antidepressants: MAO-inhibitors** | N06AF04 | Tranylcypromine |
|  | N06AG02 | Moclobemide |
| **Antidepressants: Tricyclics** | N06AA01 | Desipramine |
|  | N06AA02 | Imipramine |
|  | N06AA03 | Imipramine oxide |
|  | N06AA04 | Clomipramine |
|  | N06AA05 | Opipramol |
|  | N06AA06 | Trimipramine |
|  | N06AA09 | Amitriptyline |
|  | N06AA10 | Nortriptyline |
|  | N06AA12 | Doxepin |
| **other Antidepressants** | N06AX03 | Mianserin |
|  | N06AX05 | Trazodone |
|  | N06AX06 | Nefazodone |
|  | N06AX11 | Mirtazapine |
|  | N06AX12 | Bupropion |
|  | N06AX14 | Tianeptine |
|  | N06AX22 | Agomelatine |
|  | N06AX26 | Vortioxetine |
| **First generation Antipsychotics** | N05AB02 | Fluphenazin |
|  | N05AB03 | Perphenazin |
|  | N05AB10 | Perazin |
|  | N05AD01 | Haloperidol |
|  | N05AD06 | Bromperidol |
|  | N05AD07 | Benperidol |
|  | N05AF01 | Flupentixol |
|  | N05AF05 | Zuclopenthixol |
|  | N05AG01 | Fluspirilen |
|  | N05AG02 | Pimozid |
|  | N05AL03 | Tiaprid |
| **Second generation Antipsychotics** | N05AE03 | Sertindol |
|  | N05AE04 | Ziprasidon |
|  | N05AH02 | Clozapin |
|  | N05AH03 | Olanzapin |
|  | N05AH04 | Quetiapin |
|  | N05AH05 | Asenapin |
|  | N05AL01 | Sulpirid |
|  | N05AL05 | Amisulprid |
|  | N05AX08 | Risperidon |
|  | N05AX12 | Aripiprazol |
|  | N05AX13 | Paliperidon |
|  | N05AX15 | Cariprazin |
|  | N05AH01 | Loxapin |
| **Low-potency Antipsychotics** | N05AA01 | Chlorpromazin |
|  | N05AA02 | Levomepromazin |
|  | N05AD03 | Melperon |
|  | N05AD05 | Pipamperon |
|  | N05AF03 | Chlorprothixen |
|  | N05AX07 | Prothipendyl |
|  | N05CM22 | Promethazin |
| **Anxiolytics** | N05BA01 | Diazepam |
|  | N05BA02 | Chlordiazepoxide |
|  | N05BA03 | Medazepam |
|  | N05BA04 | Oxazepam |
|  | N05BA05 | Potassium clorazepate |
|  | N05BA06 | Lorazepam |
|  | N05BA08 | Bromazepam |
|  | N05BA09 | Clobazam |
|  | N05BA11 | Prazepam |
|  | N05BA12 | Alprazolam |
|  | N05BA16 | Nordazepam |
| **Benzodiazepine hypnotics and sedatives** | N05CD01 | Flurazepam |
|  | N05CD02 | Nitrazepam |
|  | N05CD03 | Flunitrazepam |
|  | N05CD05 | Triazolam |
|  | N05CD06 | Lormetazepam |
|  | N05CD07 | Temazepam |
|  | N05CD08 | Midazolam |
|  | N05CD09 | Brotizolam |
|  | N05CD11 | Loprazolam |
| **Z-substances** | N05CF01 | Zopiclone |
|  | N05CF02 | Zolpidem |
