# Supplementary material for: Reductions in inpatient and outpatient mental health care in germany during the first year of the COVID-19 pandemic – What can we learn for a better crisis preparedness?
Source: Eur Arch Psychiatry Clin Neurosci. 2024 Oct 2;274(8):2037–46. doi: 10.1007/s00406-024-01909-6 (PMC11579190; doi:10.1007/s00406-024-01909-6)
Supplement: Supplementary file 2 — Supplementary Material 2 [file 406_2024_1909_MOESM2_ESM.docx]

1. **Supplement Figure**

**
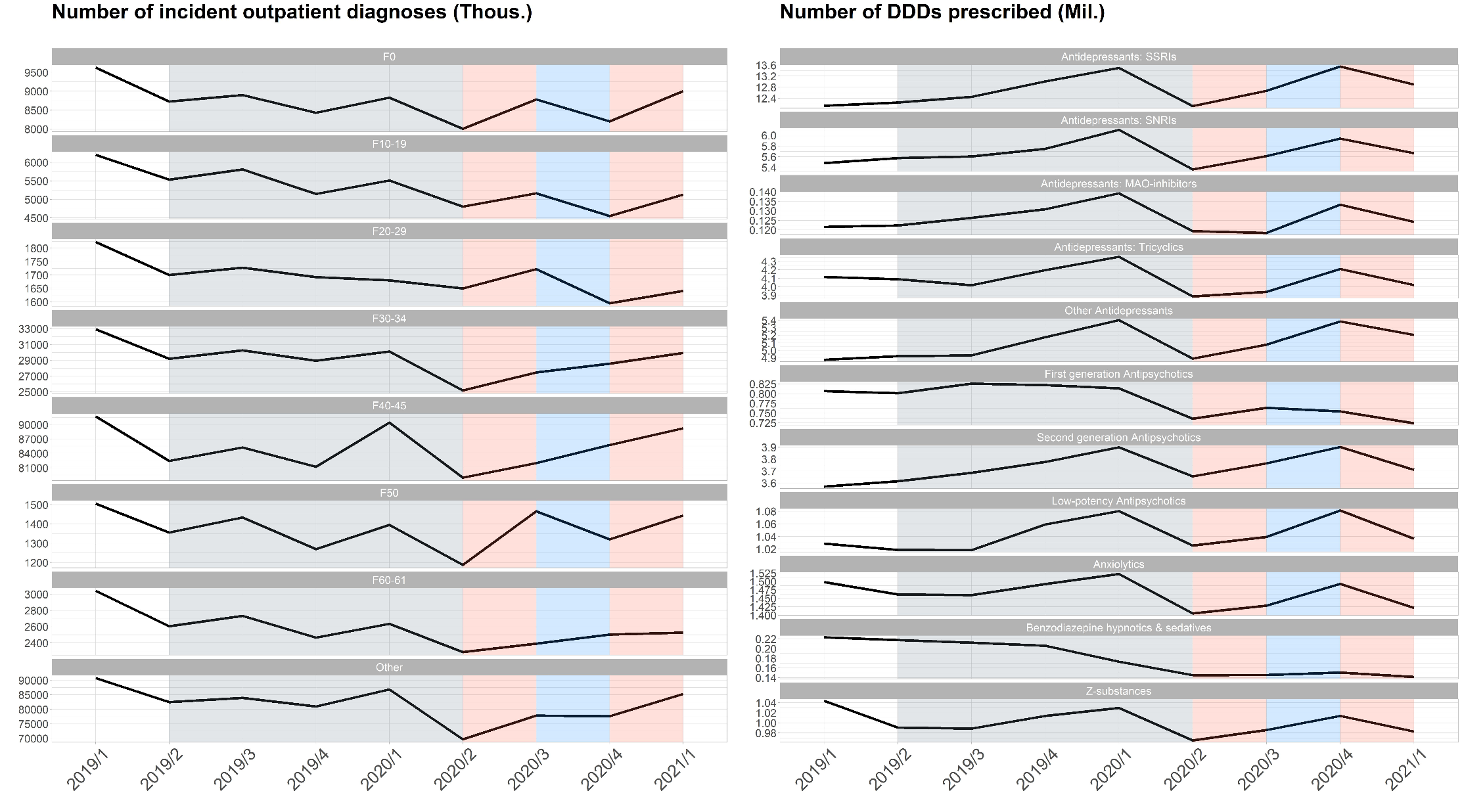
**

Number of incident outpatient diagnosis stratified by diagnostic group and prescriptions of psychotropic drugs by substance group
