# Supplementary material for: Reductions in inpatient and outpatient mental health care in germany during the first year of the COVID-19 pandemic – What can we learn for a better crisis preparedness?
Source: Eur Arch Psychiatry Clin Neurosci. 2024 Oct 2;274(8):2037–46. doi: 10.1007/s00406-024-01909-6 (PMC11579190; doi:10.1007/s00406-024-01909-6)
Supplement: Supplementary file 3 — Supplementary Material 3 [file 406_2024_1909_MOESM3_ESM.docx]

1. **Supplement Table**

Supplement Table: Risk ratios, p-values and confidence intervals taken from the time series forecast model for both of data subsets (AOK PLUS= Saxony only, BKK = entire Germany)

|  | Dataset AOK-PLUS | | | | | | | | Dataset BKK | | | | | | | |
| --- | --- | --- | --- | --- | --- | --- | --- | --- | --- | --- | --- | --- | --- | --- | --- | --- |
| **Outcome** | **Lockdown 03-05/2020** | | | | **Lockdown 12/2020-02/2021** | | | | **Lockdown 03-05/2020** | | | | **Lockdown 12/2020-02/2021** | | | |
|  | estimate | p-value | Lower CI | Upper CI | estimate | p-value | Lower CI | Upper CI | estimate | p-value | Lower CI | Upper CI | estimate | p-value | Lower CI | Upper CI |
| **Inpatient Care** |  |  |  |  |  |  |  |  |  |  |  |  |  |  |  |  |
| Total no. of admissions | 0,77 | 0,027 | 0,61 | 0,97 | 0,65 | <0.001 | 0,58 | 0,72 | 0,76 | 0.001 | 0,63 | 0,9 | 0,77 | <0.001 | 0,71 | 0,83 |
| Mean length of stay | 1,06 | 0,612 | 0,84 | 1,34 | 0,86 | 0,48 | 0.58 | 1.29 | 0,88 | 0,56 | 0,58 | 1,33 | 0,99 | 0,967 | 0,67 | 1,47 |
|  |  |  |  |  |  |  |  |  |  |  |  |  |  |  |  |  |
| **Day Clinic Care** |  |  |  |  |  |  |  |  |  |  |  |  |  |  |  |  |
| Total no. of admissions | 0,54 | 0,005 | 0,36 | 0,83 | 0,44 | <0.001 | 0,36 | 0,53 | 0,62 | 0,295 | 0,25 | 1,51 | 0,31 | <0.001 | 0,19 | 0,5 |
| Mean length of stay | 0.39 | 0.015 | 0.19 | 0.83 | 0.59 | 0.02 | 0.32 | 0.91 | 0,61 | 0,163 | 0,31 | 1,22 | 0,59 | 0,17 | 0,29 | 1,25 |
|  |  |  |  |  |  |  |  |  |  |  |  |  |  |  |  |  |
| **outpatient care** |  |  |  |  |  |  |  |  |  |  |  |  |  |  |  |  |
| No. of incident cases | 0.81 | 0.003 | 0,7 | 0,93 | 0.92 | 0.313 | 0,78 | 1,08 | 0.83 | <0.001 | 0,75 | 0,92 | 0.91 | 0.103 | 0,82 | 1,02 |
| No. of patients with psychotherapy | 1,16 | 0.01 | 1,04 | 13 | 1,09 | 0,109 | 0,98 | 1,23 | 0.93 | <0.001 | 0,9 | 0,96 | 1 | 0.971 | 0,97 | 1,03 |
| Total sum of DDDs subscribed | 1.03 | 0,536 | 0,93 | 1,14 | 0,94 | 0,24 | 0,85 | 1,04 | 0.95 | 0.009 | 0,91 | 0,99 | 1,03 | 0,035 | 1 | 1,07 |
