# Supplementary figures and images for: Reductions in inpatient and outpatient mental health care in germany during the first year of the COVID-19 pandemic – What can we learn for a better crisis preparedness?
Source: Eur Arch Psychiatry Clin Neurosci. 2024 Oct 2;274(8):2037–46. doi: 10.1007/s00406-024-01909-6 (PMC11579190; doi:10.1007/s00406-024-01909-6)

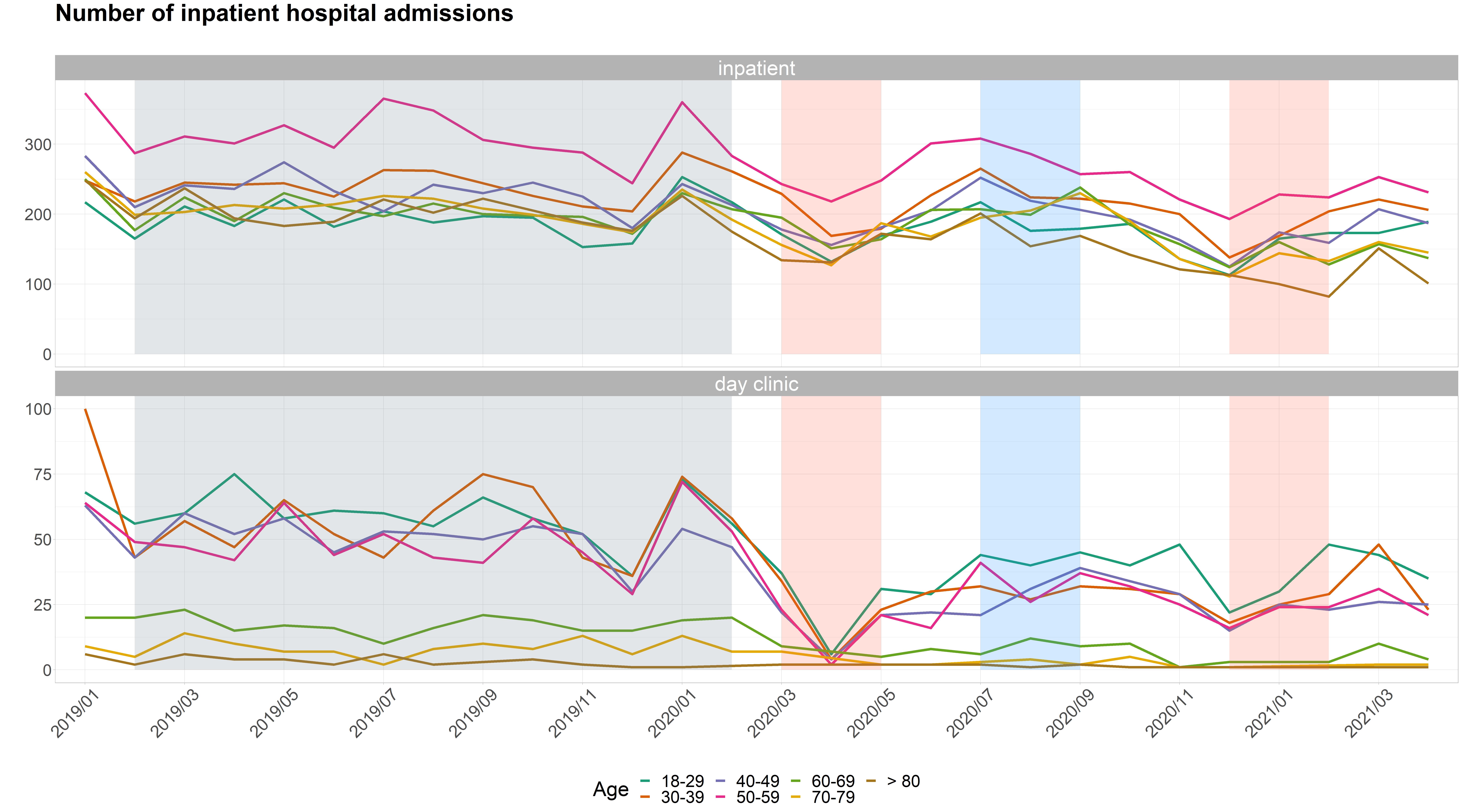

Supplement: Supplementary file 5 — Supplementary Material 5 [file 406_2024_1909_MOESM5_ESM.png]

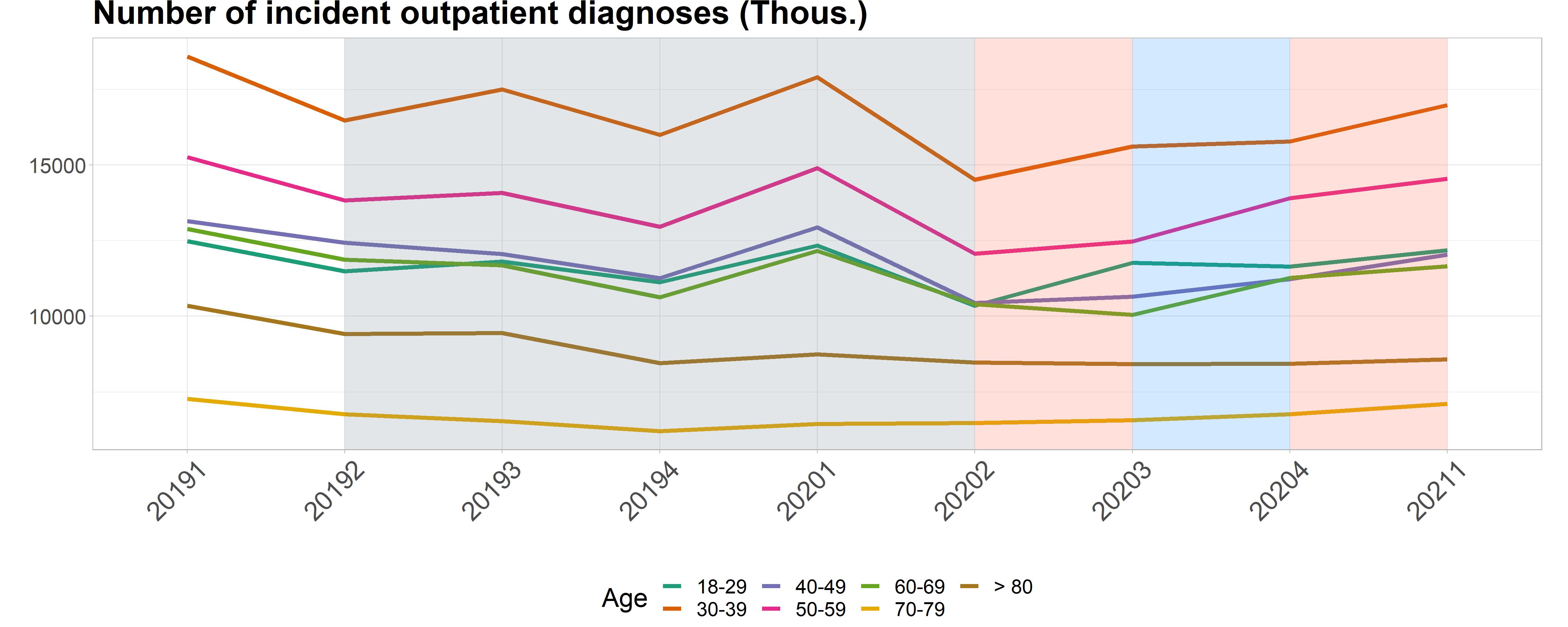

Supplement: Supplementary file 6 — Supplementary Material 6 [file 406_2024_1909_MOESM6_ESM.png]

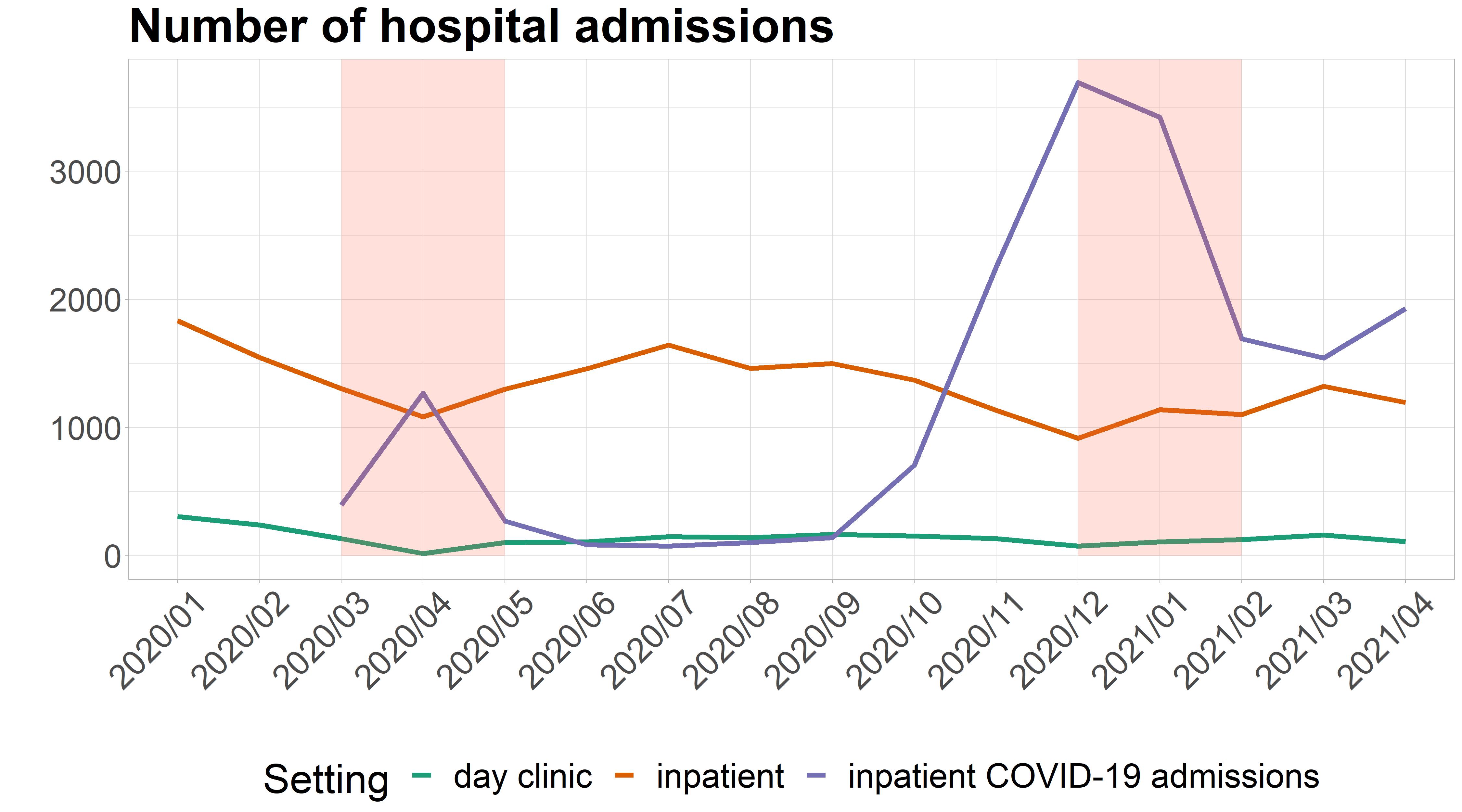

Supplement: Supplementary file 7 — Supplementary Material 7 [file 406_2024_1909_MOESM7_ESM.png]
