# Supplementary material for: Reductions in inpatient and outpatient mental health care in germany during the first year of the COVID-19 pandemic – What can we learn for a better crisis preparedness?
Source: Eur Arch Psychiatry Clin Neurosci. 2024 Oct 2;274(8):2037–46. doi: 10.1007/s00406-024-01909-6 (PMC11579190; doi:10.1007/s00406-024-01909-6)
Supplement: Supplementary file 4 — Supplementary Material 4 [file 406_2024_1909_MOESM4_ESM.pdf]

Prof. Dr. Andrea Schmitt

European Archives of Psychiatry

and Clinical Neurosciences

Dresden and Mainz, 26.01.2024

Dear Prof. Schmitt,

We wish to submit the enclosed manuscript entitled 'Reductions in Inpatient and Outpatient Mental Health Care in Germany during the First Year of the COVID-19 Pandemic – What can we Learn for a Better Crisis Preparedness ' to be considered for publication in the *European Archives of Psychiatry and Clinical Neurosciences.*

In this study, we examined changes in the utilization of inpatient and outpatient mental health services during the lockdown and the inter-lockdown phases of the COVID-19 pandemic in 2020 using routine data. For this purpose, we used a large health insurance routine dataset with data from 162,905 inpatients and 2,131,186 outpatients with mental disorders from all regions of Germany and chose a time series-based forecasting approach.

Important results are:

- For the two lockdown phases in 2020 we observed a 24-28% decrease in inpatient admissions and a 44-61% decrease in day clinic admissions. The length of stay decreased significantly for day clinics, but not for inpatients.
- Outpatient services showed a significant reduction in incident diagnoses, thus significantly reduced numbers of patients newly admitted to mental health outpatient services. There was no significant change in the number of patients receiving psychotherapeutic services or in prescriptions of psychotropic drugs.
- Overall, there was no complete recovery in utilization between the lockdown phases in 2020.

We consider the study to be relevant for the broad readership of the *European Archives of Psychiatry and Clinical Neurosciences* for the following reasons:

- Only few studies documented the changes in mental health care services during the COVID-19 pandemic comprehensively, most of them from the United Kingdom and Italy. For many other regions of Europe either only studies from limited regional provider networks exist, like for mental health inpatient services in Germany, or no studies were published at all, like for outpatient services in Germany. We think it is of high relevance to analyze these regional

changes during the pandemic in order to be able to learn from them and be prepared for future crises.

- We are using a large dataset comprising data from all over Germany. In contrast to some earlier studies, our analyses are based on a time series model, which was tested using inferential statistics. This means that longer-term changes in utilization are also included without distorting the results.
- WHO and OECD recommend infrastructures for health care monitoring and oversight as important building blocks for resilient health care systems in times of crises. Regarding the significant reductions in mental health service utilization that we observed in our study during the COVID-19 pandemic's first year, our results strongly support these recommendations. We therefore hope that the publication of our study in the *European Archives of Psychiatry and Clinical Neurosciences* can promote a debate about a better surveillance of mental health services in Europe.

With best regards,

Prof. Dr. Jochen Schmitt
Center for Evidence-Based Healthcare,
University hospital Carl Gustav Carus,
TU Dresden

Prof. Dr. Klaus Lieb
Department of Psychiatry and Psychotherapy,
University Medicine Mainz

Dr. Fabian Baum
Center for Evidence-Based Healthcare,
University hospital Carl Gustav Carus,
TU Dresden

Hauke Felix Wiegand, MD/PhD
Department of Psychiatry and Psychotherapy,
University Medicine Mainz
